# Between domestication and civilization: the role of agriculture and arboriculture in the emergence of the first urban societies

**DOI:** 10.1007/s00334-019-00727-4

**Published:** 2019-04-20

**Authors:** Dorian Q. Fuller, Chris J. Stevens

**Affiliations:** 1 Institute of Archaeology, University of London, 31-34 Gordon Square, London, WC1H 0PY UK; 2 School of Archaeology and Museology, Peking University, Beijing, 100871 China; 3 School of Archaeology and Museology, Northwest University, Xi’an, Shaanxi 710069 China

**Keywords:** Arboriculture, Near East, *Ficus*, *Olea*, *Phoenix*, *Vitis*, China, *Ziziphus*, *Amygdalus*, *Armeniaca*

## Abstract

The transition to urbanism has long focused on annual staple crops (cereals and legumes), perhaps at the expense of understanding other changes within agricultural practices that occurred between the end of the initial domestication period and urbanisation. This paper examines the domestication and role of fruit tree crops within urbanisation in both Western Asia and China, using a combination of evidence for morphological change and a database that documents both the earliest occurrence of tree fruit crops and their spread beyond their wild range. In Western Asia the domestication of perennial fruit crops likely occurs between 6500 bc and 3500 bc, although it accompanies a shift in location from that of the earliest domestications within the Fertile Crescent to Mesopotamia, where the earliest urban societies arose. For China, fruit-tree domestication dates between ca 4000 and 2500 bc, commencing after millet domestication and rice domestication in Northern and Southern China, respectively, but within the period that led up to the urban societies that characterised the Longshan period in the Yellow River basin and the Liangzhu Culture in the Lower Yangtze. These results place the domestication of major fruit trees between the end of the domestication of staple annual crops and the rise of urbanism. On this basis it is argued that arboriculture played a fundamental role within the re-organisation of existing land use, shifting the emphasis from short-term returns of cereal crops into longer term investment in the developing agricultural landscape in both Western and East Asia. In this respect perennial tree crops can be placed alongside craft specialisation, such as metallurgy and textiles, in the formation of urban centres and the shaping the organisational administration that accompanied the rise of urbanism.

**Electronic supplementary material:**

The online version of this article (10.1007/s00334-019-00727-4) contains supplementary material, which is available to authorized users.

## Introduction

The transformation of hunter-gather societies into the World’s earliest civilizations has long fascinated scholars. Gordon Childe (1936) divided this transformation into two stages: the Neolithic Revolution and the Urban Revolution. For Childe, domestication was a conscious and rapid process that fuelled the Urban Revolution, allowing settlements to grow to a previously unprecedented size (Childe 1950). Agricultural surpluses allowed the creation of “resident specialists who were themselves released from food-production” (Childe 1950, p 8) and able to engage in a range of new technologies, from specialist metallurgy to large scale textile production and to writing and administering the organisation that these new systems required. Hence the social impacts of domestication and agriculture were realized with the scaling up that occurred with urbanization, as larger concentrations of populations, including growing numbers of non-farming specialists and growing trade networks, were supported (Marston 2017; Scott 2017). The largest early agricultural villages throughout the Old World had populations in the hundreds (e.g. Kuijt 2000; Zhang and Hung 2008; Shelach and Teng 2013; Birch-Chapman et al. 2017). As the Neolithic progressed, settlements in the thousands arose (e.g. Moore et al. 2000; Shelach 2002; Birch-Chapman et al. 2017), but it was only through the processes associated with urbanization that early cities with populations in tens of thousands emerged (Dumper and Stanley 2007; Liu 2007; Algaze 2008; Liu and Chen 2012, p 282). However, Neolithic urbanization encompassed not just larger settlements, but an increased number of smaller associated settlements, some just 1 ha villages, as illustrated by regional survey data from Mesopotamia (Algaze 2008) and the northern Chinese provinces (Wagner et al. 2013).

The aim of this paper is to examine the scaling up of the agricultural systems that emerged following domestication, to those that supported some of the world’s earliest urban societies within Western Asia after 4000 bc and China after 2500 bc. The centrality of staple cereal crops to underpinning early state formation has often been emphasized (Steensberg 1989; Miller and Wetterstrom 2000; Algaze 2008; Scott 2017), fostering the development of writing, administrative structures, and increasingly hierarchical social systems (Steensberg 1989; Scott 2017). However, other factors potentially contributing to the process of agricultural change that led to urbanism have been potentially overlooked. In particular, playing a potentially major role in this development was the domestication of perennial crops: tree-fruits and vines (Miller 1991; Miller and Wetterstrom 2000; Weiss 2015). As Miller and Wetterstrom (2000, p 1,126) conclude “orchard crops began to make a noticeable contribution to the diet” at ca 3500 bc in Western Asia.

Here we consider the role of several major perennial fruit-producing plants, the origins of arboriculture as such, as part of the process of agricultural change that led to urbanism within three of the original centres of ancient civilization: in Western Asia, the middle to lower Yellow River Valley in northern China, and the Yangtze River Delta in eastern China. We consider the location and timing of their domestication through both the existing literature and the first appearance of domesticates within the archaeobotanical record. In addition we consider the general changes in agriculture associated with crop diversity as seen through the database alongside that for the role of annual and perennial crops in general.

## Comparing arboriculture, cereal domestication and urbanism

Recent work has shown that many of the assumptions and theories concerning the domestication of plants and animals are incorrect and domestication is better understood as a process of gradual evolution rather than rapid revolution. Domestication has been documented for an increasing number of species, utilizing chronologically sequenced and quantified records of morphological change through the examination of preserved remains of plants and animals themselves (Fuller et al. 2014; Larson and Fuller 2014; Zeder 2016; Allaby et al. 2017). Initial Neolithic domestications can be seen as protracted, unconscious evolutionary transformations, starting when the wild progenitors of domesticated cereals and pulses were first taken into cultivation. These actions, grouped collectively under the term pre-domestication cultivation, comprised clearance, tillage, and sowing, and gave rise to morphological changes that spread through cultivated populations over the course of a few millennia (domestication) (Fuller 2007; Fuller et al. 2014; Allaby et al. 2017), but that would have been invisible within a single human generation.

The investigation within this paper into the domestication of a number of major perennial fruit-yielding trees, shrubs and vines (here, collectively referred to as arboreal domesticates) is conducted through a similar lens to that applied to annual crops. We then compare timelines of morphological change and the geographical spread in arboriculture for individual species, alongside evidence for the relative exploitation of various cereal and pulse crops, and human population proxies.

The major domestication traits for fruit-trees in part relate to fruit size and edibility; including reduced astringency, seedlessness, and generally less stringy, softer, more palatable fruit (Janick 2005); and in part to modes of pollination, length of juvenile phases, and cloning (Miller and Gross 2011). Most significantly for some species, stone size and shape also changed, which can potentially be tracked from archaeobotanical material (e.g. Terral et al. 2010; Liphschitz et al. 2013; Pagnoux et al. 2015; Dighton et al. 2017; Fuller 2018).

Today many fruit trees and vines are grown through either grafting a cutting onto an established rootstock or planting cuttings directly. Such practices produce clones, whereas the planting of stones or seeds will produce genetic variants of both parents. In this light, given that morphological changes, as seen for cereals, are evident in the arboreal domesticates for which we have data, then early cultivation must have included the planting of stones and seeds. The patterns of change seen in stone size and shape support a protracted gradual morphological evolution for arboreal domesticates (see Fuller 2018), similar to that seen for the domestication of annual crops (see Fuller et al. 2014). Such evidence corroborates an initial emphasis during the domestication process on sexual reproduction (see Goldschmidt 2013; Weiss 2015) through the planting of stones/seeds, while simultaneously implying a slow rise in the importance of perennial crops. This theory is contrary to the inference of Zohary et al. (2012) that perennial domestication was a rapid and conscious process in comparison to annual cereals and pulses, and that it involved primarily vegetative propagation. However, selection processes, in terms of human action and its interaction with fruit tree genetics, are still poorly understood.

Seed size increase during domestication is a general trait recorded for many species, including cereals and vegetables (Kluyver et al. 2013, 2017). In seed-grown annuals, such changes can be associated with the creation of a level playing field through cultivation, potentially including burial (Harlan et al. 1973; Fuller 2007; Gegas et al. 2010; Fuller and Stevens 2017). How these explanations translate to increased size seen in fruit stones, pips etc. is less clear. In most fruits, increased seed length is achieved with minimal increase in overall embryo volume. Therefore, Fuller (2018) suggests that the lengthening of perennial fruit stones increases the total fruit flesh with minimal additional investment in seed volume, and thus infers some conscious element of selection for more edible matter in fruits which in turn increases seed length and the length:width ratio. Alternatively, as with cereals, if a number of stones, pips, or seeds were planted at the same time, then those with larger (more elongate) seeds that produce stronger seedlings might be selected through the removal of smaller, less well-established seedlings. However, experimental observations of correlations of seed/stone size and shape and seedling characteristics in fruits are still needed to test these theories.

**Fig. 1:**
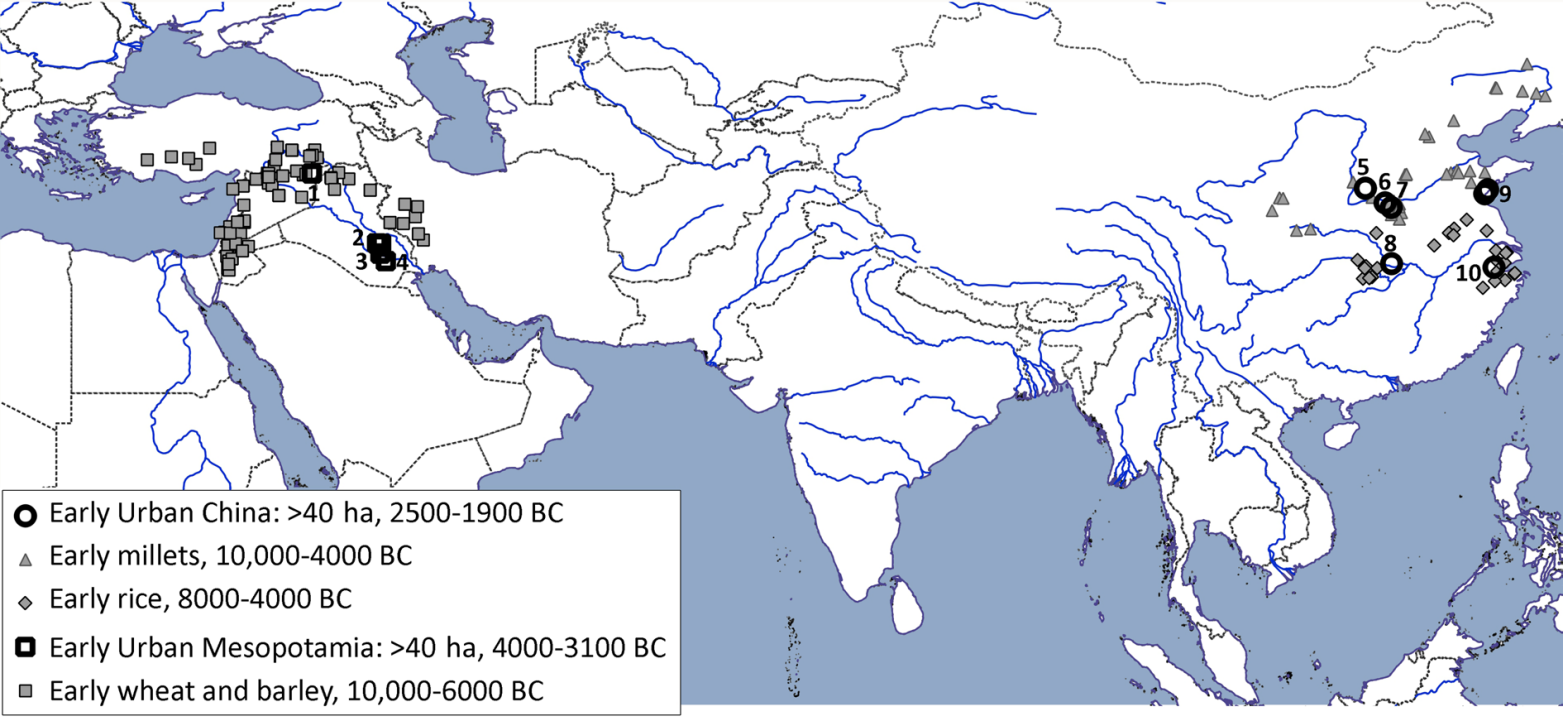
Map of early centres of domestication compared to centres of urbanization for the Near East, Northern China and the Yangtze Valley. This plots sites with early cultivation evidence (10000–6000 bc for the Near East; 8000–4000 bc for North and South China, data from ESM Tables S1, S2, S3) with a selection of early urban sites, over 40 ha (from the fourth millennium bc for Mesopotamia; from 2500 to 1900 bc for China). Early urban centres in relation to zones of agricultural origins in West Asia and China. Selected primary urban centres, > 40 ha: (1) Tell Brak, (2) Tel Delhim and Tell al-Hayyad, (3) Uruk (Warka), (4) Eridu, (5) Taosi, (6) Erlitou, (7) Wangchenggang, (8) Shijiahe, (9) Liangchengzhen and Yaowangcheng, (10) Liangzhu

Despite these issues, the metrical data (Fuller 2018), combined with database evidence for both the earliest appearance and increased presence of fruit-tree crops, lead to the hypothesis that their domestication and incorporation into pre-existing patterns of agricultural land-use was a contributing factor in urbanization in Western Asia and China. While each region contained centres of domestication and subsequent urbanization (Maisels 1998), these processes are more spatially distinct in Mesopotamia than was likely the case in the Yellow River or Yangtze River (Fig. 1). Hence the Liangzhu Culture broadly matches the same area in the Lower Yangtze for which at least one trajectory of rice domestication has been mapped (Fuller et al. 2009, 2016). Likewise the Longshan Culture arose in the middle to lower Yellow River Valley (Henan, Shandong) and the Guanzhong Plain (Lower Wei Valley), which comprise at least three of the five possible centres for millet domestication (Stevens and Fuller 2017). Both China and West Asia have rich archaeobotanical records that document the processes of domestication of annual grain crops, including cereals and pulses. For both regions the process of cereal domestication spans around 2,000–3,000 years. In Western Asia the process of domestication for cereal and pulse crops terminates around 7500–7000 bc (Fuller et al. 2018), whereas for China the completion of cereal domestication appears to have occurred largely around 4500–4000 bc (cf. Stevens and Fuller 2017).

For China, the beginnings of urban society appear to grow directly from the end of the initial domestication period with perhaps only less than two millennia separating the end of cereal domestication (ca 4000 bc) from the initiation of urbanisation (ca 2500 bc) (Liu and Chen 2012). Conversely, the founding crops of West Asian civilizations, wheat and barley, were not domesticated where the first evidence for urbanization is found. Rather they spread from the core areas of the northern and southern Levant and/or eastern Fertile Crescent into the middle to lower Euphrates valley after ca 7000 bc, with urbanisation occurring around three millennia later, after ca 4000 bc, in the middle and lower Euphrates and Tigris valleys (Maisels 1998; Algaze 2008).

## Materials and methods

The assessment of the early cultivation and exchange of selected perennial fruits is based on a database compilation of crop archaeological occurrences, the Old World Crops Archaeobotanical Database (OWCAD), developed by the ComPAg (Comparative Pathways to Agriculture) project at University College London (UCL). The database covers Asia, Africa, and more selectively Europe, from the terminal Pleistocene to historic times. It provides an index of the primary literature from which other information, such as quantitative data on occurrence or metrics, can be derived. In total, the database includes ~ 2,300 archaeological sites with archaeobotanical data, some broken down into multiple phases. These data have previously been used to chart the spread of cereals between East and West Asia (Stevens et al. 2016), and within East and Southeast Asia (Stevens and Fuller 2017). Here we utilize a subset of data representing Western Asia and China, and associated peripheral regions. The Western Asia data includes 489 sites/phases (based on median calibrated radiocarbon ages) (ESM Table S1). The Chinese sites dating predominately to between 7000 and 1 bc are divided into two sub-regions: a northern Chinese region centred on the Yellow River Basin (Henan, Inner Mongolia, Jilin, Liaoning, Qinghai, Shaanxi, Shandong, Shanxi and Zinjiang), comprising 434 sites/phases (ESM Table S2), and a Southern Chinese region, corresponding mainly to the Yangtze basin (Guangdong, Guangxi, Guizhou, Hong Kong, Hubei, Hunan, Jiangsu, Jiangxi, Shanghai, Sichuan, Tibet, Yunnan and Zhejiang) and comprising 115 sites/phases (ESM Table S3). In preparing maps we have included the additional occurrences of fruit taxa outside these regions, including in Egypt, Aegean Europe, Western Central Asia, and South Asia, and the sites plotted on each of the maps of early fruit occurrence are also provided in supplementary tables (ESM Tables S4–S10).

These data provide a basis for assessing the distribution of fruits in space (via maps) and time, and in respect to their inferred wild native ranges. Temporal patterns are considered in terms of ubiquity across sites in time slices, which allow comparison of general trends in the occurrence of these species (following Miller 1988; Weber 1991). As a baseline comparison we show the same statistics for cereals and major pulses. Morphological change in West Asian crops and Chinese rice, and soybean, have been published elsewhere (Fuller et al. 2014; Allaby et al. 2017) and are summarized from those sources in this paper. Datasets tracking the morphological change in some of the fruit species considered here, namely peach, date and olive, have also been published elsewhere (Fuller 2018).

## Results: West Asia—the domestication and dispersal of fig, grape, olive, and date

**Fig. 2:**
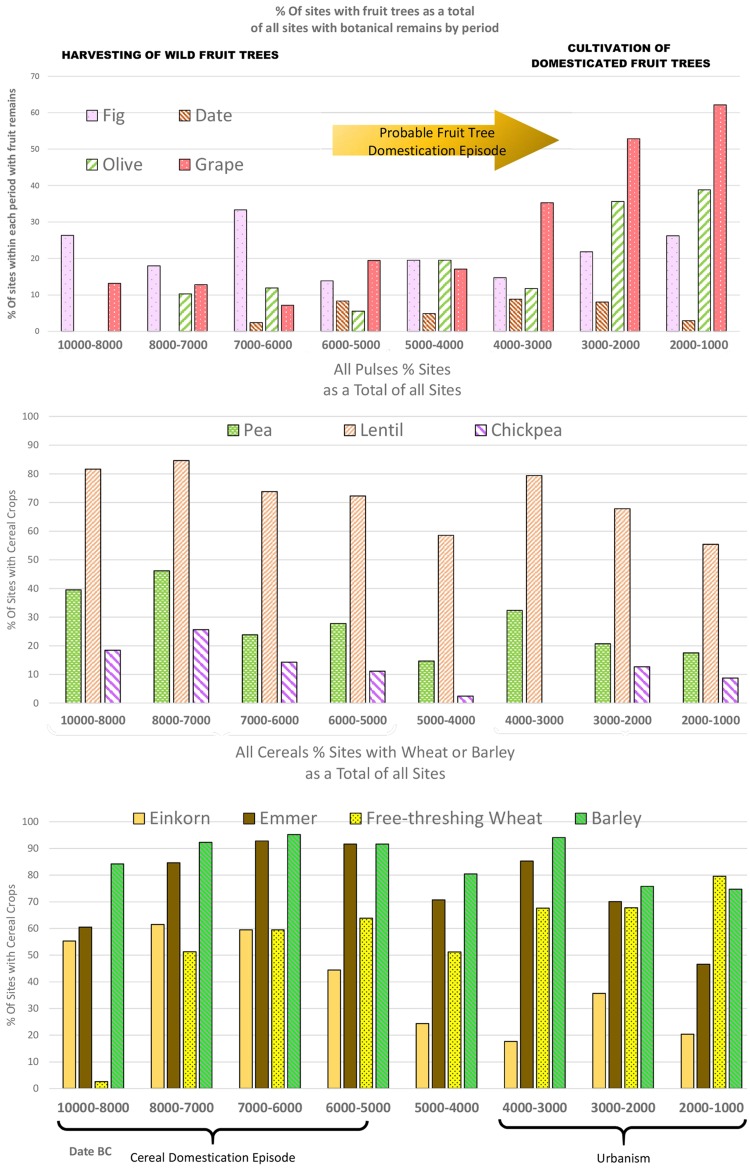
Percentage of West Asian sites divided from 10000 to 8000 bc and into 1 millennium time blocks from 8000 to 1000 bc with (top) remains of fig, date, olive and grape, (centre) remains of leguminous crops—pea, lentil, chickpea and (bottom) remains of cereal crops—einkorn, emmer, free-threshing wheat, barley. Note the decline of hulled wheats to free-threshing wheats and the increase in grape and olive

The geographical distribution of archaeobotanical records for remains of all four species indicates that their earliest exploitation began within the areas of their plausible wild distributions in the pre-Pottery Neolithic, first occurring outside these ranges after the fifth millennium bc (the Chalcolithic), and particularly from the fourth millennium bc. The rising importance of these species overall is evidenced from regional ubiquity data with archaeobotanical occurrences of stones/pips increasing in the fourth millennium bc, and is especially marked for the grape and olive, both of which increase subsequently in the third millennium bc (Fig. 2). Dates are never particularly frequent, but this is likely due to their limited native range and cultivation falling to the south of Mesopotamia. These patterns in fruit occurrence need to be considered alongside a chronology of urbanization, which in the first part of the fourth millennium bc (4000–3600 bc) saw the emergence of half a dozen sites from 25 to 80 ha, with the massive growth of Warka to ~ 250 ha by 3100 bc (Algaze 2008). With respect to annual crops, two trends are worth noting. The first is that some of the fall-off seen within annual pulses is because as these crops spread out from West Asia the package broke apart, with chickpea, for example, being a rare component in Iran (cf. Stevens et al. 2016). The second is that there is a genuine decline in hulled wheats, particularly einkorn, during the lead into urbanism with a slight but notable increase in free-threshing wheat, a pattern that was noted in an earlier study by Miller (1991).

### Fig (*Ficus carica*)

The earliest evidence of consumption of fig comes from Pre-Pottery Neolithic A sites located in the Jordan Valley stretching north into the Upper Euphrates, and it is plausible that cultivation had already commenced at this time. Domestication of this species likely comprised two pathways: one involving wild recessive mutations, the other parthenocarpy (fruit development without fertilization). In the former, recessive genes confer elongated carpels, with a long style, to female flowers, allowing larger fleshy fruits to develop, whilst restricting the ability of pollinating wasps to lay their eggs. These produce large sweet fruits with crunchy “seeds” (so-called Smyrna figs). Within this domestication process, female plants would need to have been cultivated alongside hermaphrodite plants (caprifigs) with both male and short-style female flowers, expressing the dominant genotypes and harbouring egg-laying fig wasps. Within the second pathway, parthenocarpic mutants produce seedless fruits without being fertilized (common figs or Adriatic figs). Kislev et al. (2006) reported charred examples of such seedless fruits from Gilgal (ca 9300 bc). However, Denham (2007) cautions that such mutants, occurring naturally in wild stands, may have been preferred by gatherers, and fig seeds appear widespread at other Pre-Pottery Neolithic A and early Pre-Pottery Neolithic B sites, e.g. Jericho, Gesher, Mureybit III, Tell Qaramel, Jerf el Ahmar, and D’jade (Willcox et al. 2008; Table 1 in Fuller et al. 2012), and it is possible that cultivation of Smyrna-type figs had begun alongside early sedentary Pre-Pottery Neolithic A villages cultivating cereals and pulses. However, wild figs were likely still present within natural riverine gallery vegetation (cf. Bogaard et al. 2017), and collection entirely from the wild at this date cannot yet be ruled out.

Some fig cultivars are triploid, and others tetraploid, suggesting a role of polyploidy within both wild populations and early cultivars that may have potentially conferred improved traits to their fruits (Falistocco 2016). Given the ease and rapidity with which fig shrubs can establish in or around middens, they may even have become part of the human-constructed ecological niches of early Pre-Pottery settlements in the Mediterranean Levant.

**Fig. 3:**
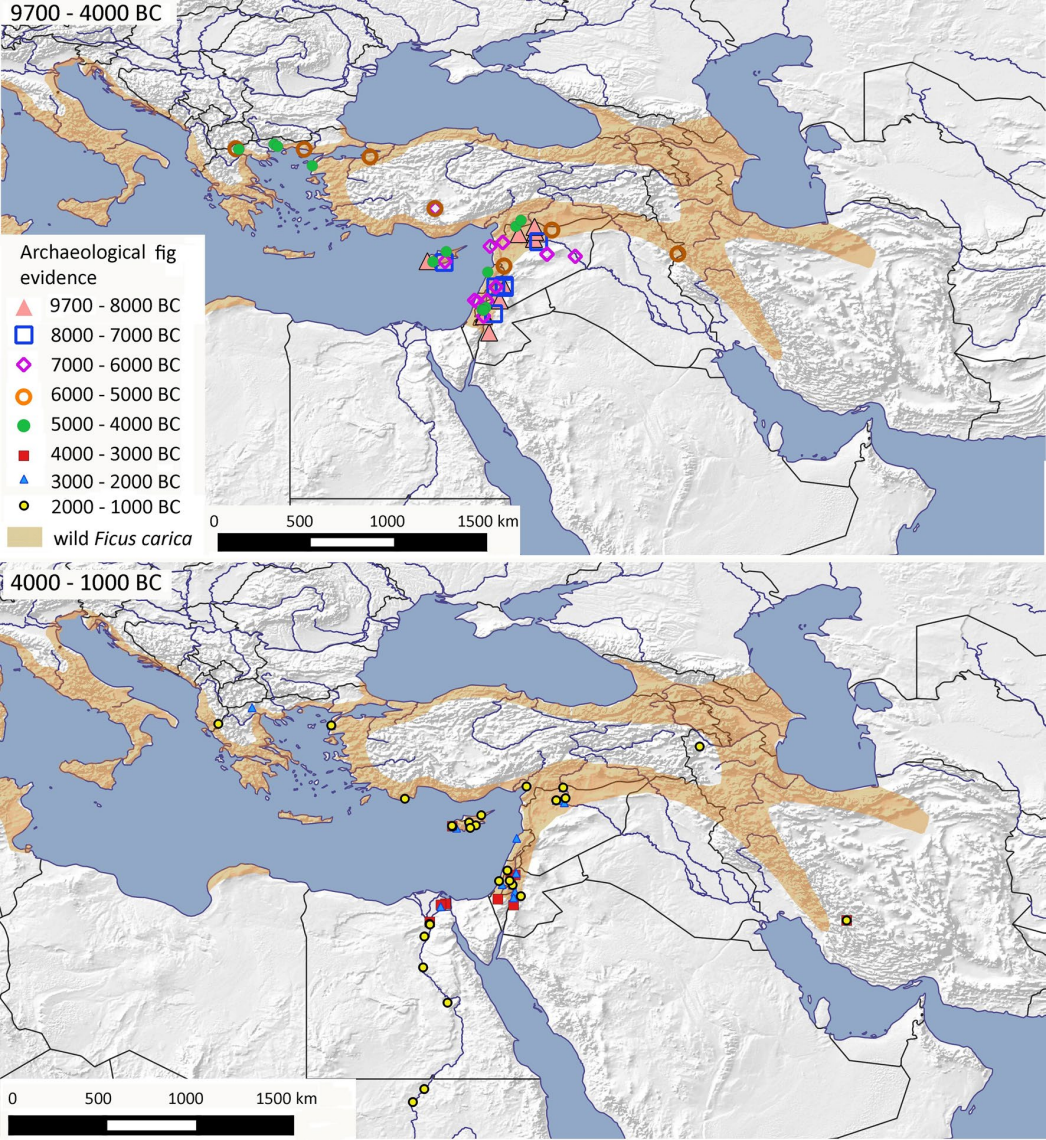
Archaeobotanical finds of fig within West Asia, North Africa and adjacent regions (mainly *Ficus carica* but potentially also *Ficus sycomorus* especially in North Africa) against the wild distribution for common fig (*Ficus carica*). Sites are shown in millennium blocks from 9700 to 4000 bc above, and from 4000 to 1000 bc below (ESM Table S4)

Archaeological finds of figs that fall outside their projected wild zone (Fig. 3; cf. map 17 in Zohary et al. 2012) are first seen in the fourth millennium bc, including southern Iran. Fourth millennium bc finds in the Nile Delta presumably represent the dispersal of cultivated *F. carica*, although it remains unclear where in the Nile Valley native *F. sycomorus* was originally distributed, or how early it came under cultivation (most likely through vegetative propagation). However, such finds only begin in the third millennium bc (Zohary et al. 2012, p 130) and its native pollinator wasp (*Ceratosolen arabicus*), assumed to have been once present in Egypt, has long since been extirpated and persists today only in sub-Saharan Africa. *F. sycomorus* figs are sometimes parthenocarpic, although more often they are notched or scraped to induce fruit set (Harlan 1986). Both *F. carica* and *F. sycomorus* are clearly differentiated in Egyptian art (and later literature) from at least the Old Kingdom, c. 2500 bc (Brewer et al. 1994), and cuneiform sources record the cultivation of assumingly common figs from a similar date in Mesopotamia (Postgate 1987).

### Grape (*Vitis vinifera*)

**Fig. 4:**
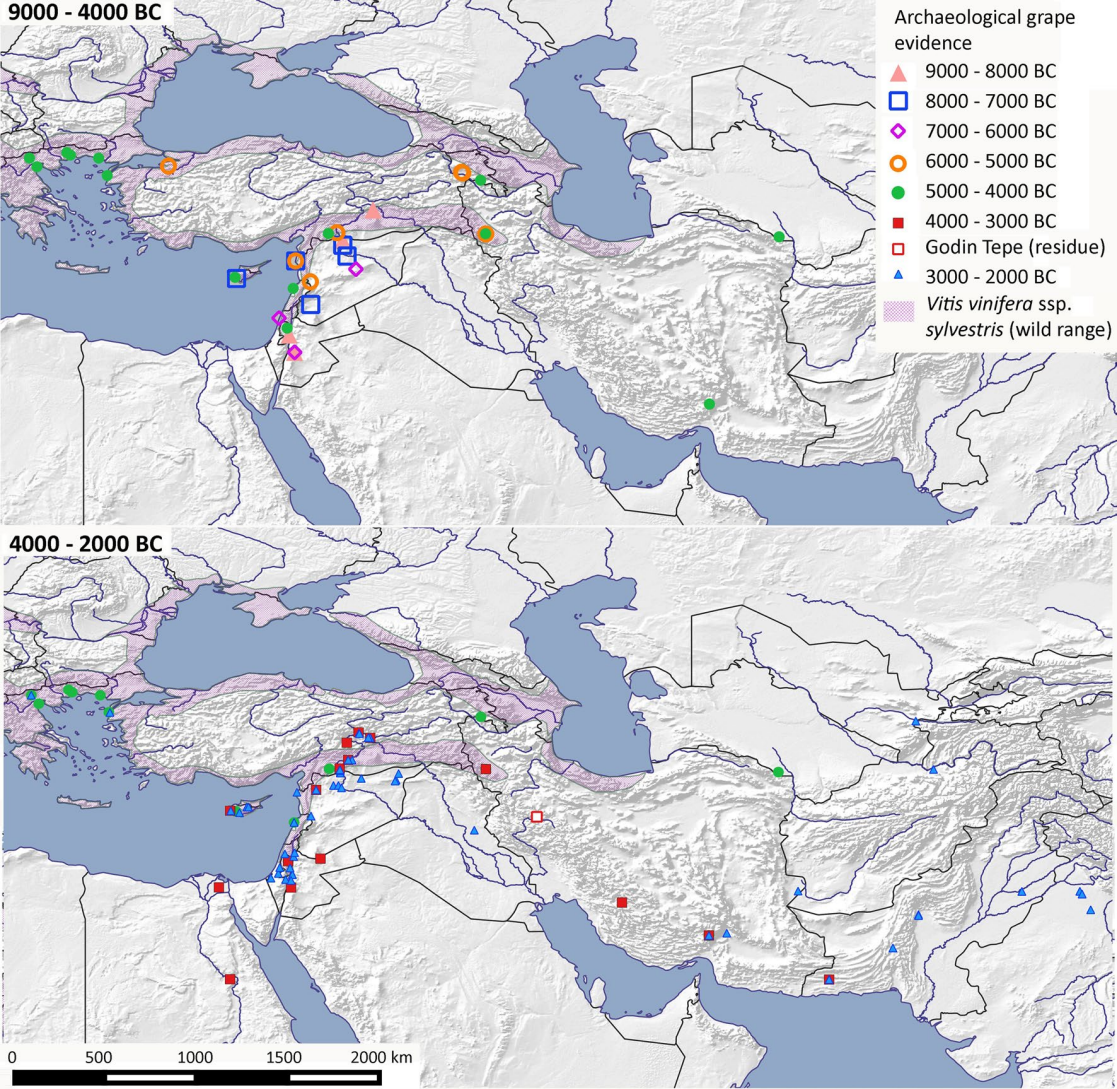
Archaeobotanical finds of grape (*Vitis vinifera*) within West Asia, North Africa and adjacent regions against the wild distribution for grape (*Vitis sylvestris*). Sites are shown in millennium blocks from 9000 to 4000 bc above, and from 4000 to 1000 bc below (ESM Table S5)

Similar to common fig, early finds of olive and grape appear restricted to the Mediterranean Levant, where they form part of the wild flora (Fig. 4). For grape, the earliest finds mainly come from the Jordan Valley and the northern Fertile Crescent, i.e. the Upper Tigris Basin, dating to the ninth millennium bc, although two pips of *Vitis* are recorded from Ohalo II (ca 21000 bc) on the Sea of Galilee (Kislev et al. 1992). By the eighth millennium bc, grapes are more widespread in intervening areas of the Levant and Cyprus. Whether wild grapes were part of the native Cyprus flora, or came with the pre-domestication cultivation package that included crops, livestock, and commensals, is unknown. Claims have been made for grape domestication and the origins of wine in the early sixth millennium bc from the Caucasus (McGovern 2003; McGovern et al. 2017). However, grape macroremains from the earliest periods have yet to be recovered, and evidence for early wine making is based on tartaric acid from pottery residues. However, tartaric acid is commonly found in other fruits (cf. Barnard et al. 2011), e.g. wild crab apples, hawthorn, or *Sorbus*, the last notably being cited by Virgil (Georgics Lib. III, 379–380) as consumed by Scythians as a fermented alcoholic imitation of wine.

The early archaeological finds of grape from the Levant and northern Fertile Crescent more strongly support initial cultivation and domestication in this region, where a diverse array of annual crops was already long established. Late Neolithic finds of grape (c. 4300 bc) from northern Greece are likely related to their collection from the wild (Valamoti et al. 2007; Valamoti 2015), while wine production is supported by finds of burnt pressed grapes along with ceramic residues from jars at Dikili Tash (Garnier and Valamoti 2016). Thus, we can regard exploitation of grapes for early wine production as potentially widespread throughout the area of wild grape distribution. Likewise, evidence for wine making is also evident from around 4000 bc at Areni Cave, Armenia (Barnard et al. 2011), also within the range of wild grape, although in this case it may relate to cultivated grapes.

It is also from the oak woodlands of western Asia, where wild grapes grow, that *Saccharomyces cerevisiae* originated. *Saccharomyces cerevisiae* is the key yeast responsible for turning grapes to wine, and phylogenetics suggest that bread yeasts derived from these early wines (Legras et al. 2007), while beer yeasts had other distinct origins in part (Gonçalves et al. 2016). Thus, the evolution of leavened doughs, such as those that presumably filled Uruk-era ceramic bread moulds, e.g., the bevel-rimmed bowl (Goulder 2010), represent a technological exaptation of wine fermentation. The transfer of yeasts from grape wine into cereal products including beer and leavened bread was appreciated by Miller and Wetterstrom (2000) and Sherratt (1999). Indeed, these chemical transformations of food stuffs allowed the full nutritional advantages of agriculture to be more fully realized, as fermentation made available more digestible nutrients, as well as “new forms of storage and cooking possibilities” (Miller and Wetterstrom 2000, p 1,126).

Evidence for the start of systematic cultivation remains elusive. Morphological change in terms of grape pip lengthening would certainly be indicative but may only evolve later in the domestication process, and is yet to be systematically studied for West Asian *Vitis* (see Bacilieri et al. 2017). Nevertheless, what is clear is the period when grapes expanded beyond their natural wild distribution, which can be taken as indicative of cultivation, if not domestication (Fig. 4). Grape pips, as well as grape pollen, are recorded beyond their wild range in eastern Iran by the fifth millennium bc, a finding further supported by grape pollen in Lake Zeribar, western Iran from c. 4300 bc (Miller 2008). By the end of the fourth millennium bc, grapes were present in the Egyptian Nile Valley, and probably the Indus Valley, where they became an important part of urban societies by the third millennium bc. The trade in wine can be seen to be well established in Western Asia within written sources by the third millennium bc (Postgate 1987; Tengberg 2012). Leavened breads, a probable by-product of Mesopotamian urbanisation, also likely spread along expanding trade networks, including into Egypt, as part of wider Mesopotamian cultural influence (see Wengrow 2006).

### Olive (*Olea europaea*)

**Fig. 5:**
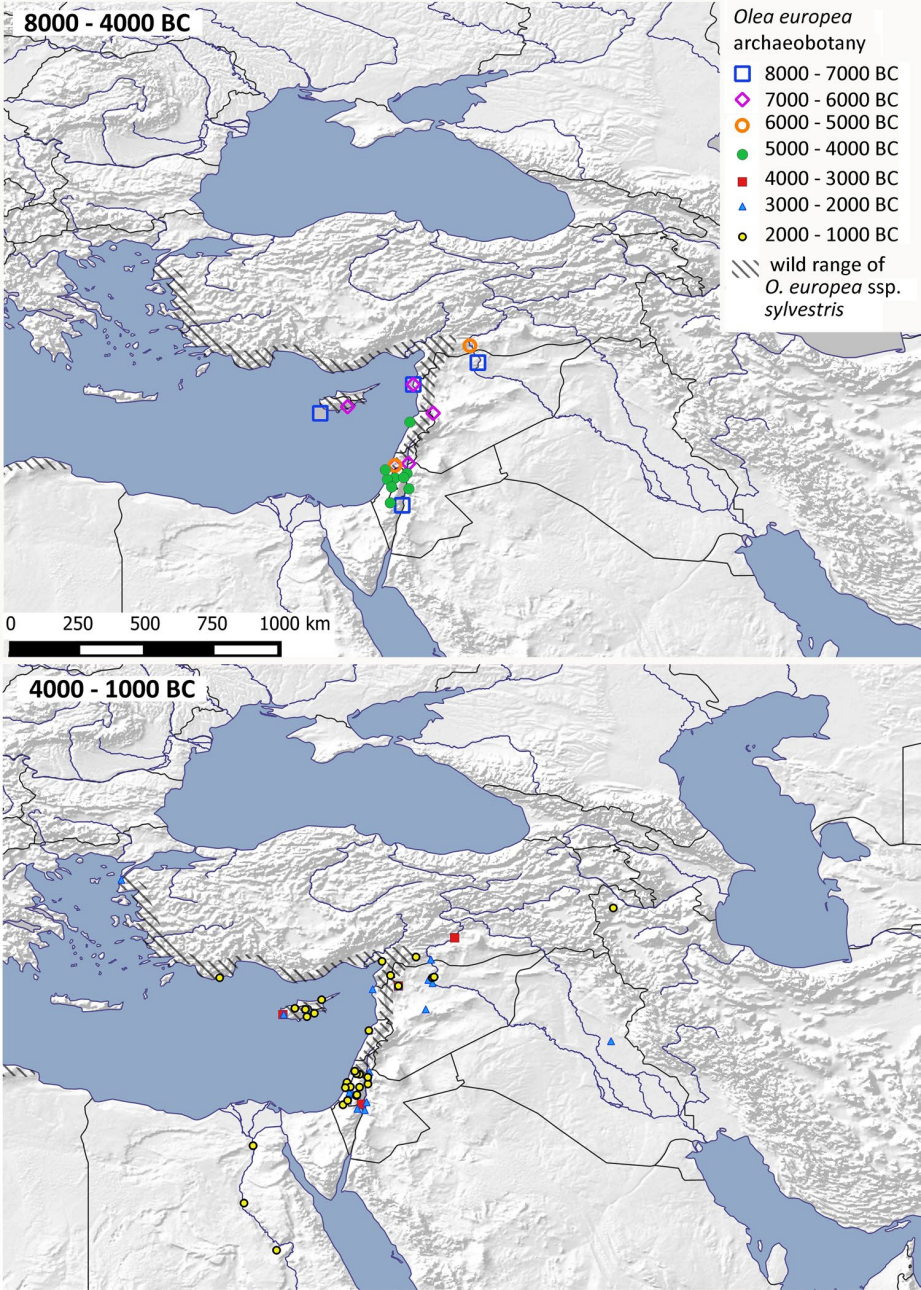
Archaeobotanical finds of Olive (*Olea europaea*) within West Asia, North Africa and adjacent regions against its wild distribution. Sites are shown in millennium blocks from 8000 to 4000 bc above, and from 4000 to 1000 bc below (ESM Table S6)

Early olive finds are frequent throughout the Levant and Cyprus, generally within their expected wild range, which, while similar to that of grape, is more restricted to the fringes of the Mediterranean (Fig. 5). Olive stones from Levantine sites show increased length indicative of cultivation from at least 4500 bc and continuing into the early Bronze Age, after 3500 bc (Dighton et al. 2017; Fuller 2018). Earlier evidence for pressing olives comes from northern Jordan ca 5200 bc (Dighton et al. 2017) and coastal Israel, although these may be of wild olive (Tengberg 2012), suggesting cultivation was perhaps established earlier, through a mixture of seed propagation (to account for seed size increase) and vegetative propagation. Ultimately, vegetative propagation came to dominate and is key to maintaining varieties. Earlier finds dating from the eighth millennium bc at Halulua on the Upper Euphrates (Willcox 1998), lie only 100 km beyond the present wild range, and might indicate a wider wild distribution than present (Fig. 5). However, finds from fourth millennium Hassek Höyük, southern Turkey (Gregor 1992), some 150–200 km beyond present wild distributions, are more plausibly associated with its dispersal as a cultivar. In the third millennium bc, additional finds come from east of the wild range, including one in eastern Iraq, probably representative of trade. By the third millennium bc, olive trees were being managed and pressed for oil at Ebla, Syria, as seen through textual evidence (Tengberg 2012), and archaeological evidence in parts of Greece and the Aegean (Margaritis 2013). Olives appear to be established later than grapes in Egypt, possibly only in the New Kingdom when *shaduf* irrigation facilitated the expansion and diversification of garden and orchard cultivation (Eyre 1994). Earlier finds may indicate importation of olives to Egypt during the Middle Kingdom, from ca 2000 bc (Caracuta et al. 2018).

### Date palm (*Phoenix dactylifera*)

**Fig. 6:**
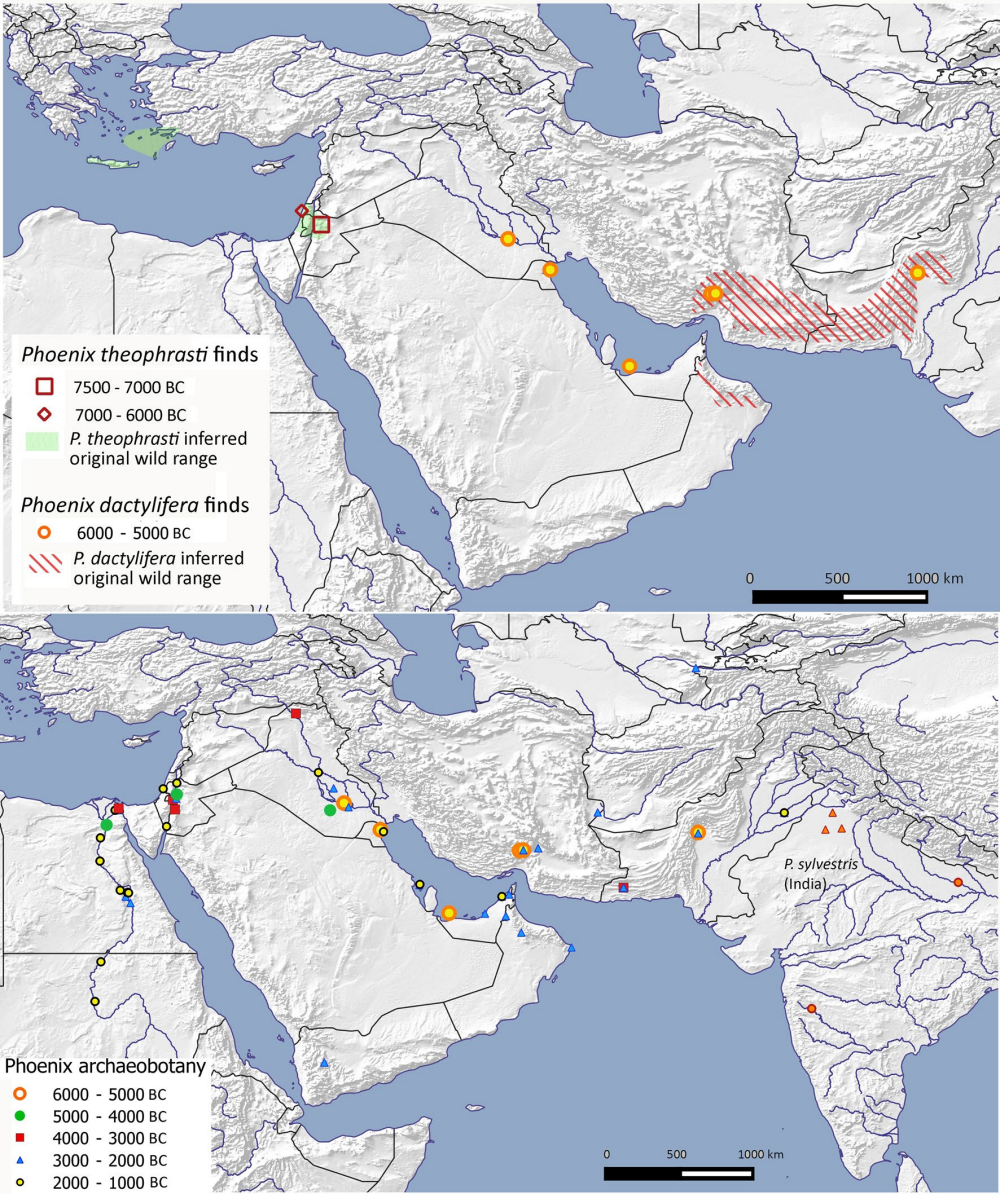
Archaeobotanical finds of *Phoenix dactylifera* within West Asia, North Africa and adjacent regions against the inferred wild range for wild *Phoenix dactylifera* and *P. theophrasti*. Sites are shown in millennium blocks with finds of *Phoenix dactylifera* and *P. theophrasti* from 7500 to 5000 bc above, and all finds from 6000 to 1000 bc below (ESM Table S7)

Date palm differs from those crops discussed above in originating probably from the Arabian Peninsula, with the wild distribution potentially stretching across the Gulf of Oman into Pakistan (Fig. 6), but seemingly less likely into North Africa (Flowers et al. 2019; cf. Gros-Balthazard et al. 2016). This is in agreement with Late Pre-Pottery Neolithic evidence for date palm from northern Israel, which indicates the presence of the closely related *P. theophrasti*, (Kislev et al. 2004), which explains the eighth millennium records for *Phoenix* from Ain Ghazal and Atlit-Yam in Jordan and Israel, respectively (see Rivera et al. 2014; Flowers et al. 2019). Taken together, the implication is that the wild distribution of *P. theophrasti* originally spread along the eastern edge of the Mediterranean, making it unlikely that the (early Holocene) distribution of wild *P. dactylifera* spread this far north or indeed west into Africa (contra map 17 in Zohary et al. 2012).

The earliest finds of true date, *P. dactylifera*, come from eastern Arabian sites in the sixth millennium bc, supporting Arabia as the likely origin of wild date and early cultivars, especially given the recent recognition of relict wild populations in Oman (Gros-Balthazard et al. 2017; Flowers et al. 2019). Early finds in southeast Iran and southern Pakistan potentially indicate former more easterly wild populations or perhaps early dispersal across the Persian Gulf/Gulf of Oman (Fig. 6). Finds from Eridu in Southern Mesopotamia, Teileilat Ghassul in the Jordan valley, and El Omari in the Lower Nile, indicate the age of domesticated palm moving north and west (Fig. 6; Zohary et al. 2012, p 134), suggesting cultivation probably commenced as early as ca 4500 bc (Fuller 2018). Artistic evidence, including early pictographic script, from southern Mesopotamia indicates that date palm cultivation was well-established by Late Uruk times (late fourth millennium bc) (Miller et al. 2016), and in the third millennium bc date palms are commonly referred to as a major constituent of fruit orchards and frequently associated with burials (Tengberg 2012). Measurements on date stones demonstrate that a general directional size change towards elongated date stones was still occurring after the Late Uruk period, from the third through to the first millennium bc (Fig. 3 in Fuller 2018). Despite a few Predynastic and early Dynastic reports from Egypt, date palm cultivation in Egypt and Nubia only appears well established from the Middle Kingdom onwards, becoming increasingly important from the New Kingdom period when the *shaduf* improved irrigation of gardens and orchards (Eyre 1994).

## Results: East Asia—the domestication and dispersal of peach, apricot and Chinese jujube

As in Western Asia, the earliest Chinese civilizations were centred on cereals—millets (*Setaria italica* and *Panicum miliaceum*) in the Yellow River Basin and rice (*Oryza sativa*) in the Yangtze—that were dominant in each respective region by ca 4000 bc (Liu and Chen 2012; Stevens and Fuller 2017). In terms of annual grain staples, early Chinese agriculture is less diverse than that of West Asia, although morphological changes suggest soybeans were domesticated in Central China (the Yellow River basin) during the Middle Neolithic, between 3500 and 1500 bc (Lee et al. 2011; Fuller et al. 2014), while wheat was introduced to China around 2000 bc (Stevens et al. 2016). However, it should be noted that foxtail millet became by far the most dominant crop within the Yangshao period in northern China in the period between domestication and urbanisation, while the increase in millet crops in Southern China is largely due to the expansion of millet agriculture from the Yellow River into southern parts of China predominantly after 3500 bc, where it was a minor accompaniment of rice agriculture (Stevens and Fuller 2017). Urbanisation in China was a dispersed phenomenon with some large regional centres appearing in the third millennium bc in the rice-growing Yangtze (e.g. Shijiahe, Liangzhu), but with urbanisation in the Central Plains established by ca 2000 bc at sites with *Setaria italica* as the dominant grain (e.g. Taosi and Erlitou) (Liu and Chen 2003; Liu 2007).

**Fig. 7:**
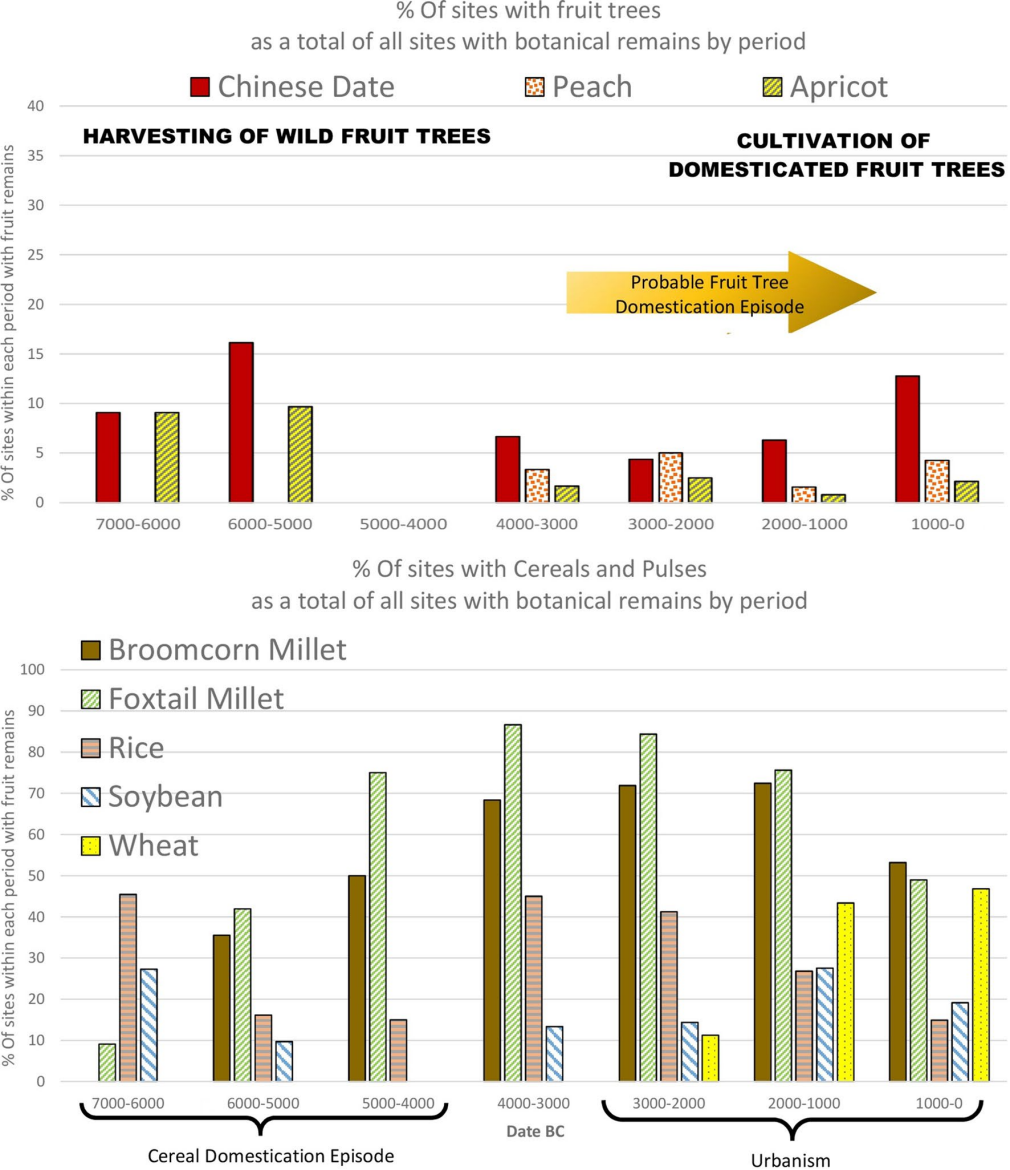
Percentage of North Chinese sites divided into 1 millennium time blocks from 8000 to 0 bc with remains of Chinese jujube, peach and apricot above and broomcorn millet, foxtail millet, rice, soybean and wheat below (ESM Table S2)

**Fig. 8:**
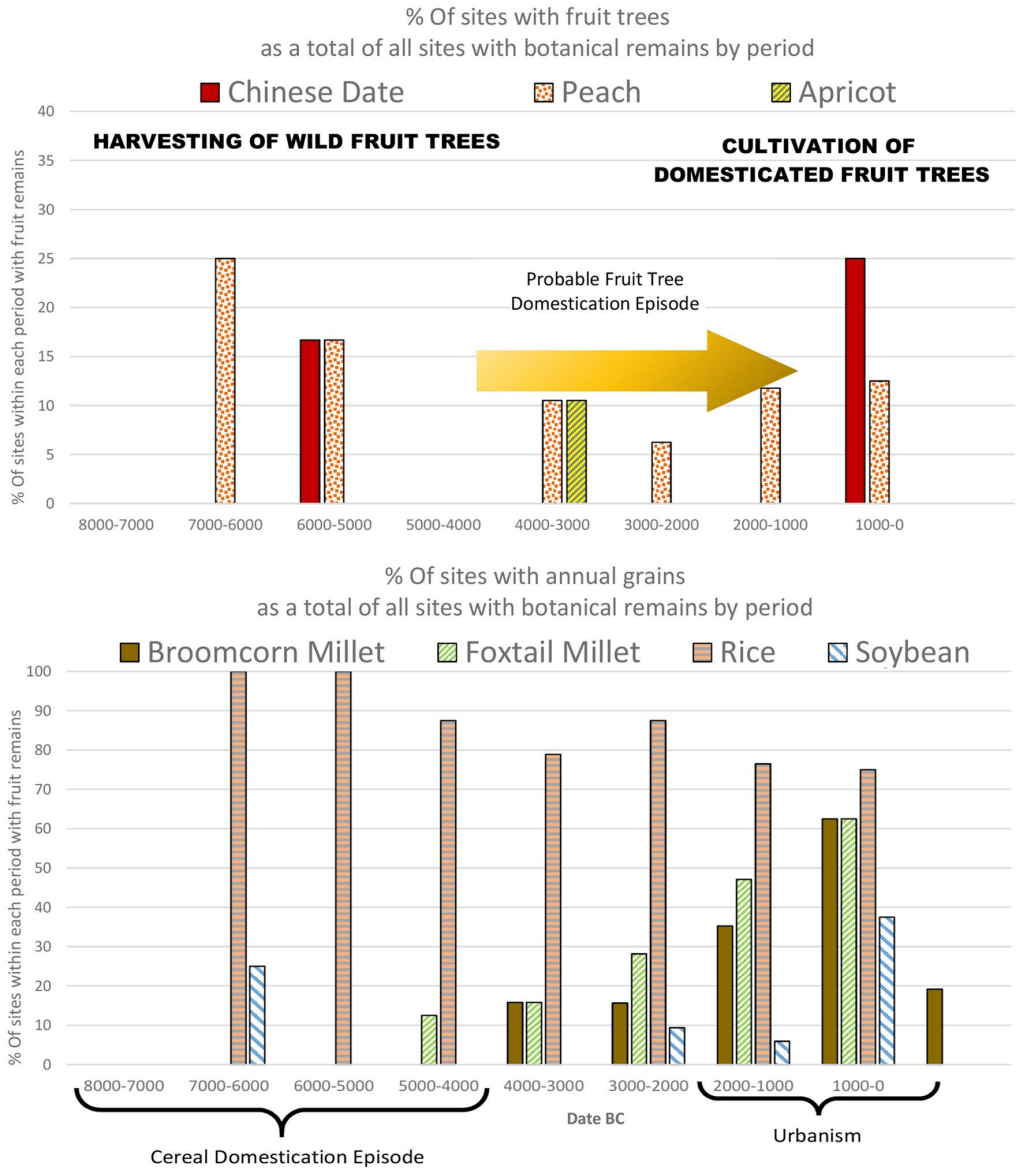
Percentage of Southern Chinese sites divided into 1 millennium time blocks from 7000 to 0 bc with remains of Chinese jujube, peach and apricot above and broomcorn millet, foxtail millet, rice, and soybean below (ESM Table S2)

The fruits we discuss are those with a good archaeobotanical record, by virtue of their hard, woody endocarps: namely peach, apricot, and jujube (or Chinese date). These three species are likely native to China and are referenced in the Late Bronze Age poems of the *Xijing* (*Shih Ching*) (see Keng 1974; Anderson 1988). While their archaeobotanical remains occur sporadically throughout the Neolithic and Bronze Age, in neither Southern (Fig. 7) nor Northern China (Fig. 8), as defined by the Qinling Huaihe Line, is there an obvious increase that signifies systematic fruit cultivation. Taken individually we can see that the record for each of the three fruit species suggests increased use and probable domestication starting in the later fourth or third millennium bc, following the prime cereal domestications, when Neolithic settlement patterns shifted towards urbanism. It is likely there was a shift from wild exploitation, alongside cereal domestication, to primarily sourcing fruit from cultivation. Such a scenario can be related to the expansion of agriculture across the Neolithic, which was accompanied by deforestation as seen through a marked decline in arboreal pollen at a regional level (see Fig. 5 in Stevens and Fuller 2017).

### Peach (*Amygdalus persica*, including *A. ferganensis*)

**Fig. 9:**
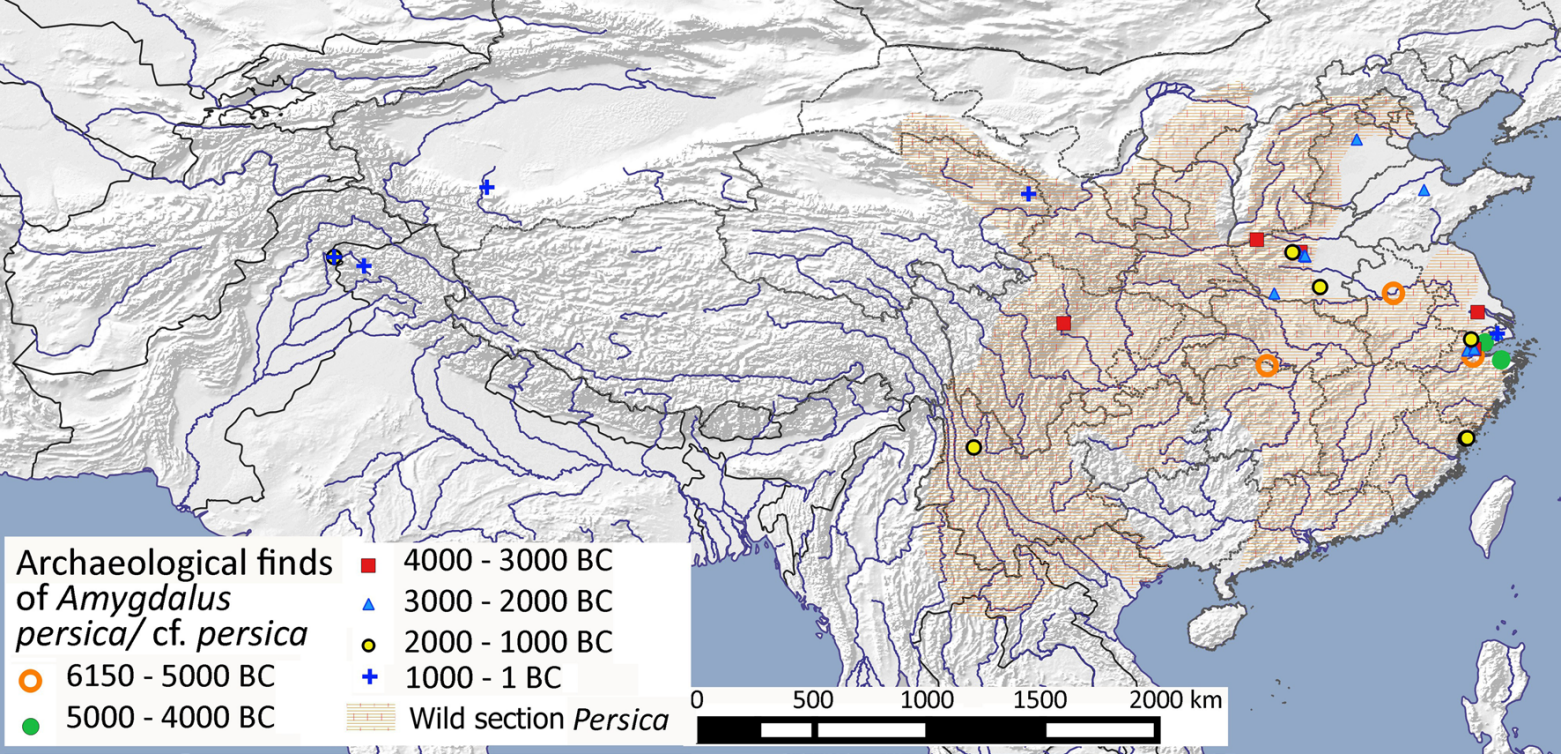
Archaeobotanical finds of *Amygdalus persica* and *A*. cf. *persica* from China and India and the inferred probable range of section Persica (ESM Table S8)

Unambiguous wild populations of *A. persica* (syn. *Prunus persica*), are not clearly documented, although many scholars have inferred Chinese origins (e.g. Li 1970; Keng 1974; Huang et al. 2008; Weisskopf and Fuller 2014a). Many also support Darwin’s conclusion that the wild ancestor of peach is nowhere to be found (Yazbek and Al-Zein 2014). Nevertheless, Pliocene fossils (> 2.6 million years old) from Yunnan (Southwest China) affirm an indigenous wild distribution in at least part of China (Su et al. 2015). One challenge is that related species, whose stones display similarly ridged or deeply rugose endocarps, occur both in wild and in semi-cultivated states, such as *A. mira* which is grown and consumed in parts of southwestern China (Sichuan and Yunnan). Other related wild peaches, including mountain peach (*A. davidiana*) and Gansu peach (*A. kansuensis*), are widely distributed across Northern China, but there are no wild peach species present today within the eastern provinces south of Shandong. Recent taxonomy groups all wild peaches into a strongly monophyletic subgeneric section Persica, distinct from the western Eurasian section Amygdalus of almonds (Yazbek and Oh 2013; Yazbek and Al-Zein 2014). The distribution of section Persica provides a broad geography from within which true wild peaches are likely to have been brought into cultivation. This range is plotted in Fig. 9, following Delplanke et al. (2016), and excluding small outlier populations on the Tibetan Plateau, and in Mongolia and Kazakhstan.

Genetic work suggests two scenarios: a single domestication followed by multiple feral peach cultivars, or multiple independent domestications giving rise to multiple observed traits (Akagi et al. 2016). This same study demonstrated hybridization between single accessions of wild *A. davidiana* and *A. mira*, and cultivated *A. persica*. This observation, together with the lack of an obvious genetic progenitor from Northern China, despite the presence of two wild peach species there today, and given that the wild peach ancestor is likely to have been geographically isolated from closely related species, would support domestication from a now extinct progenitor that came under cultivation within at least the Lower Yangtze, where the earliest archaeological evidence has been recovered, but possibly also the Huai River and/or Middle Yangtze. Under this scenario early records of peach from Northern China might represent their collection from the wild rather than early cultivation.

The earliest archaeobotanical records of peach from the lower Yangtze, Yangtze delta, Middle Yangtze, Huai Valley and Northern China date from 6000 to 3500 bc (Fig. 9). Stones of *A. persica* from around 6000 bc to ca 2300–2000 bc were studied from sites within the Lower Yangtze and Yangtze delta (Zheng et al. 2014). This study noted that stones became larger and less spherical through time, and while little change is seen between 6000–5000 bc, from 3500 to 2000 bc there is a considerable increase in size (Fig. 2 in Fuller 2018; Fig. 6 in Zheng et al. 2014). This suggests the initiation of cultivation, at least in the Lower Yangtze, began around 4000 bc, coinciding with the end of rice domestication. Genetic work also indicates a strong bottleneck in the cultivar around 3000 − 2000 bc, when compared to the wild mountain peach *A. davidiana* (Faust and Timon 1995). That peaches were under cultivation by 3000 − 2000 bc is supported not just by the increase in stone size, but also the first occurrence of peach in the Shandong region. The subsequent appearance of peach in Late Neolithic Kashmir and in Late Jomon Kyushu, Japan, during the second millennium bc, suggests translocation of the cultivated tree (Weisskopf and Fuller 2014a; Zheng et al. 2014; Stevens et al. 2016). In summary, morphological change was under way in the third millennium bc in Chinese peaches with translocation to distant regions by the second millennium bc taking place during the period of urbanization.

### Apricot (*Armeniaca vulgaris*)

As with peach, the domestication history of apricot raises a number of issues. While the wild ancestor is unknown, wild or feral populations are widely distributed not only throughout China, but also in Central Asia, the Caucasus Mountains, and along the Himalayan foothills. As long ago as 1882, de Candolle (1885, pp 215–218) argued for a Chinese origin, based on the recovery of small wild-type fruits from Henan, together with historical records showing a slow dispersal into Europe and West Asia, a clear great antiquity of use in China, and linguistic evidence including its long-established single character in Chinese, *xing* (杏), comprising the compounds for tree/wood and mouth. Genetic evidence (microsatellite markers) indicated three clusters of diversity focused on the Caucasus, eastern Central Asia, and Central China (Maghuly et al. 2005), although this study lacked systematic sampling of Chinese cultivars or wild populations. However, it seems likely domestication originated in China, with later introgression with wild populations in Xinjiang and the Caucasus (Weisskopf and Fuller 2014b), diffusing at a later date via central Asia into Eastern Europe (Maghuly et al. 2005).

**Fig. 10:**
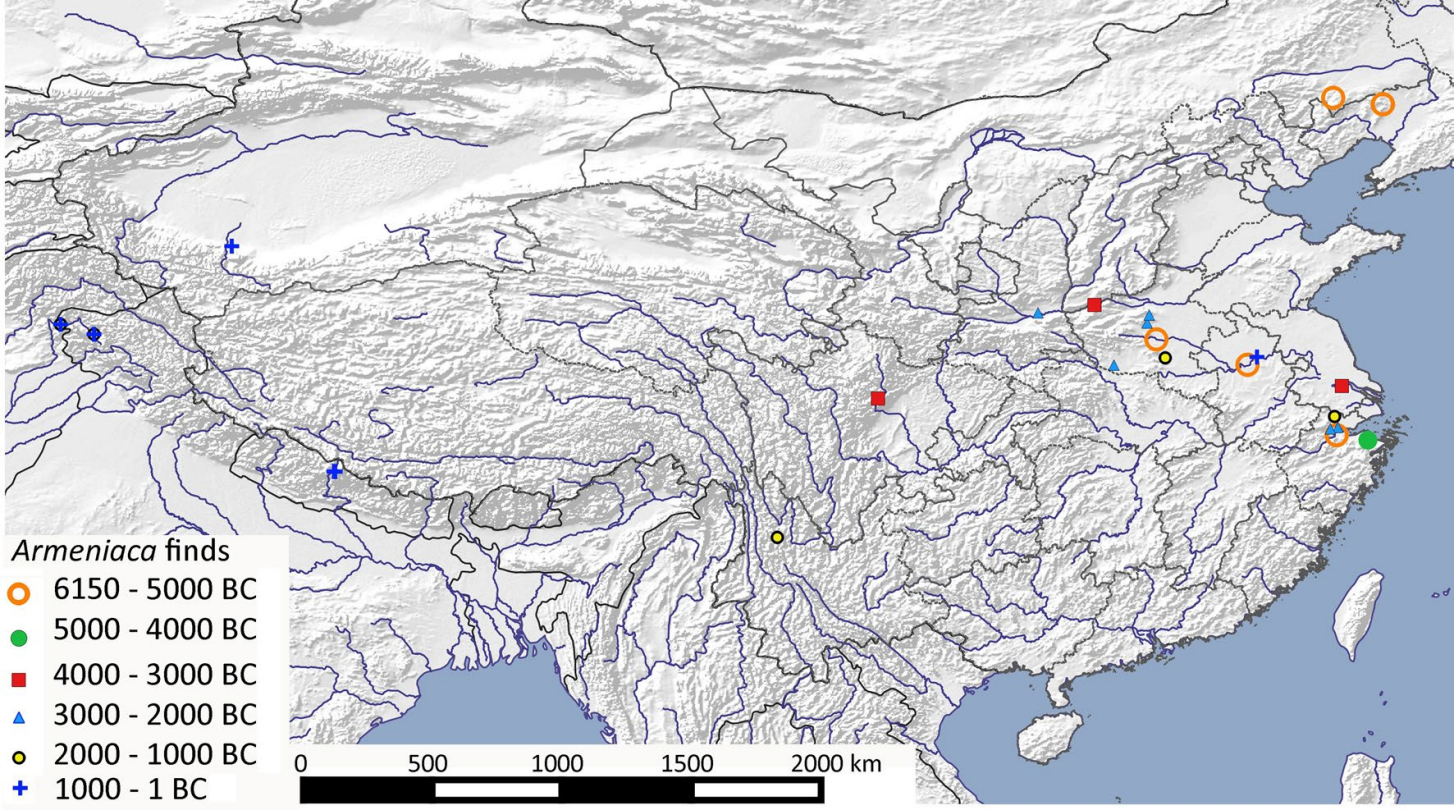
Archaeobotanical finds of *Armeniaca* from China, India and Nepal, from c. 6000–0 bc (ESM Table S9)

The earliest records for apricot in China are widely dispersed, covering northeastern China, the Huai River, and the lower Yangtze delta region (Fig. 10). Regarding wild species in China it might be noted that the *Flora of China* (Lu and Bartholomew 2003) lists a number of wild species of apricot including *Armeniaca holosericea* in north-western China, *A. hongpingensis* in Hubei and Hunan, and a number of other species distributed across Southern China. Of some interest is that the modern distributions of *A. mandshurica, A. sibirica* and wild *A. vulgaris* var. *ansu* in Northern China overlap with each other, which, given that all can readily hybridise, suggests that some may be feral populations. Today only wild *A. vulgaris* var. *ansu* is reported from Jiangsu, with no records from modern Zhejiang, which suggests, as with peach, that at least one area of likely domestication lies within the lower Yangtze or Yangtze delta, given the region’s continuous archaeobotanical record. In northern China, early records could potentially represent other wild species of *Armeniaca*, although there are no genetic studies as yet to indicate the relationship or indeed differentiation between wild species within China.

Early specimens of apricot are reported at around 4000–3000 bc from Neolithic settlements in Ukraine (see Zohary et al. 2012), but these are difficult to reconcile with the general pattern of evidence for apricot. It is possible that they represent a separate Caucasian origin, but caution is warranted given that reports of Chinese millets from Ukraine at this time are equally problematic (Motuzaite-Matuzeviciute et al. 2013; Stevens et al. 2016). We therefore conclude that cultivated apricot originates in China, and that its occurrence in Kashmir at the end of the local Neolithic during the second millennium bc marks the first dispersal of cultivated apricots outside of China (Stevens et al. 2016).

### Chinese jujube (*Ziziphus jujuba* var. *jujuba*)

There exists some confusion regarding the taxonomy of this species, and presently the two cultivars are often both assigned to *Z. jujuba* Mill. However, archaeological and genetic evidence indicates that they should be separated into two distinct species and hence domestication events: *Z. jujuba* var. *jujuba* and *Z. mauritiana* Lam. (Indian jujube). Genetic studies show clear separation between the cultivated Indian and Chinese jujube, together with its wild progenitor, sour jujube (*Z. jujuba* Mill. var. *spinosa* (Bunge) Hu ex H. F. Chow, syn. *Z. acidojujuba, Z. vulgaris* var. *spinosa*) (Ma et al. 2011). Further, recent genetic studies in China suggest that the clustering of two wild varieties of *Z. jujuba* var. *spinosa* with separate clades of cultivars might relate to two distinct domestication events (Xu et al. 2016), or two regional patterns of introgression.

**Fig. 11:**
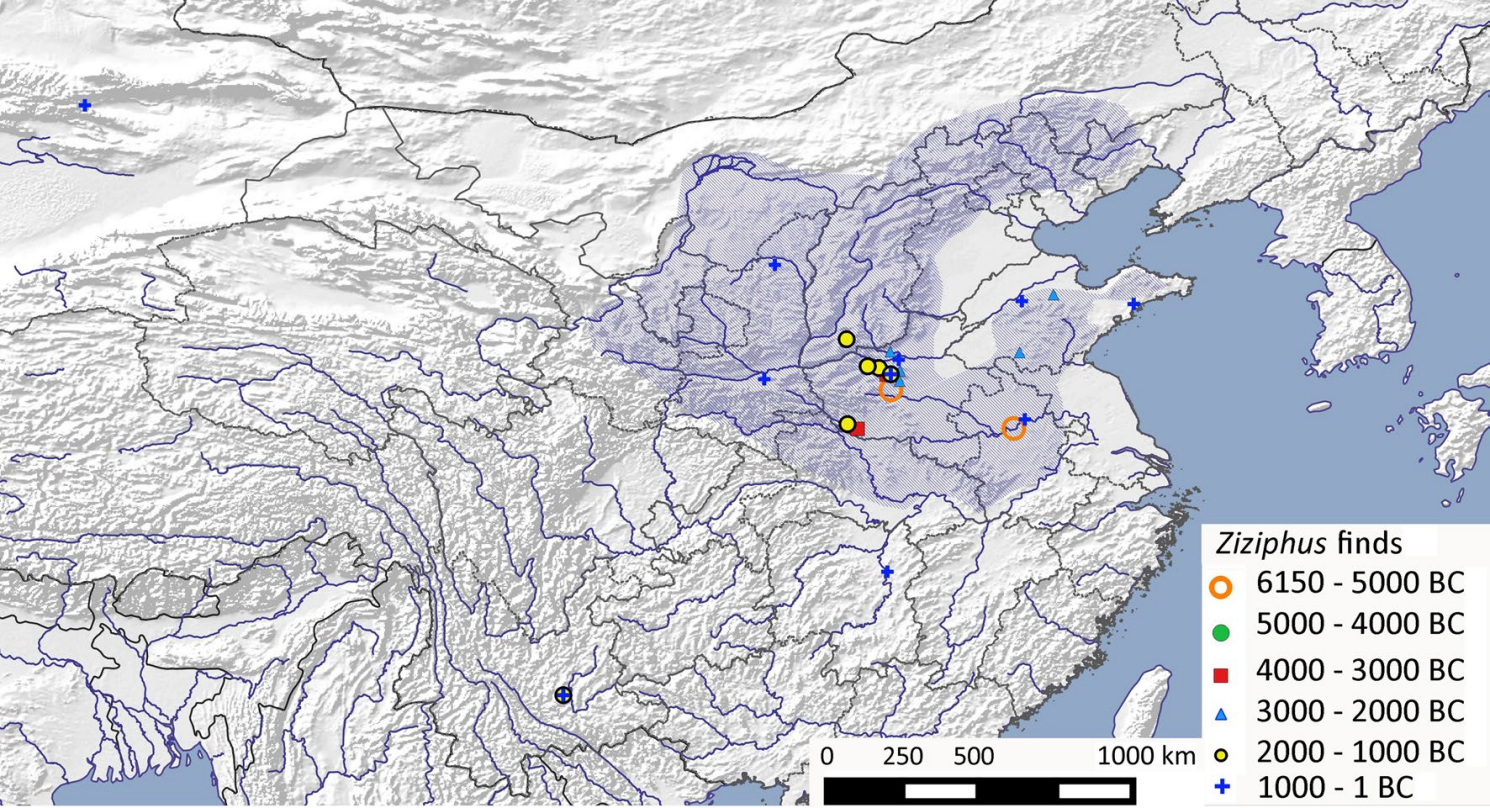
Archaeobotanical finds of *Ziziphus jujuba* var. *jujuba* (Chinese jujube) from China and the inferred probable range of wild *Z. jujuba * var. *spinosa* (ESM Table S10)

Wild jujubes are characteristic of secondary growth in the temperate deciduous broadleaf forests of Central and Northern China, from Liaoning in the east towards Gansu and Qinghai in the west (Wang 1961, pp 74–92). The wild progenitor *Z. jujuba* var. *spinosa* is widely distributed in Northern China, as well as the mountain foothills of Anhui and Jiangsu, with other congeneric species confined to southwest China (Fig. 11 shows its potential wild distribution). A number of early records exist for the Upper Huai River/Middle Yellow River, with a single early record in the Middle Huai. The continuous archaeobotanical record within this region suggests it was most likely first cultivated here alongside millet. Unlike peach and apricot, jujube is largely absent from archaeobotanical records for the lower Yangtze. Findings outside this zone, including in Yunnan, would suggest that domesticated, cultivated varieties were present by at least the second millennium bc.

Differentiation between the more globular stones of wild sour jujube and the narrower pointy stones of modern cultivars (see Liu et al. 2008, pp 137–138) allow the two to be distinguished and suggest a potential means for tracking Chinese jujube cultivation. While no such studies have been conducted for China, the identification of jujube stones as wild or cultivated stones from a number of sites does allow some preliminary observations to be made. At Xijincheng, Henan, cultivated stones were only identified from later Tang/Song dynasty deposits, with wild-type stones seen in Longshan deposits (Chen et al. 2010), while definitive cultivated stones resembling modern specimens were present in the Early Western Han period (Zhao and Wang 2016). Given the two to three millennia of morphological domestication seen for other arboreal domesticates, these data suggest that cultivation potentially begun in the Late Longshan period. Slightly elongated and acute stones from Yangshao Yuqiao, Henan (ca 3000 bc), may suggest that selection resulting from cultivation had started at this date, although the later Longshan stones from nearby Xiawu still included round wild types some 1,000 years later (Fuller and Zhang 2007). As with other trees today, Chinese jujube is reproduced through grafting, but as discussed above the observed morphological changes within the stone shape implies that until at least the Han period its cultivation was through planting the stone. Planting from seed would have also maintained high levels of variation and potential reversion to more wild-like forms via cross-pollination, and thus high variation in shape among early archaeological finds is to be expected.

## Discussion: a comparative summary of fruit tree cultivation before urbanization

The evidence presented above demonstrates how the domestication of the major fruit trees, with the possible exception of figs, likely falls within the time period that follows the domestication of annual grain crops and establishment of agriculture, but before the emergence of large urban centres of population within both regions. In his definition of the Urban Revolution, Childe (1936, 1950) laid emphasis on craft specialization, metallurgy, the trade of craft items over long distances, and the accumulation of surplus and wealth in early cities, where ruling classes lived and monumental public buildings were created. The reconsideration of fruit-tree domestication outlined above, in line with Childe’s theory of urbanization, indicate a shift from the cultivation and domestication of annual staples, which can be regarded as driven by subsistence needs centred on maintaining food security, to domestications that relate to Childe’s other aspects of subsistence, such as trade, craft production, and defining social status.

It might be noted that several authors have highlighted agricultural intensification as key to the process of urbanization (e.g. Trigger 2003; Yoffee 2005). However, isotope data from cereal grains spanning the Neolithic to Bronze Age indicate a general decline in manuring intensity in West Asia, suggesting that more extensive rather than intensive agricultural systems supported urbanism (Araus et al. 2014; Styring et al. 2017). Although the creation of irrigation systems during the fourth millennium bc in at least Southern Mesopotamia (Wilkinson and Jotheri 2019) is likely to have played a role in such changes, agricultural intensification is better demonstrated for more recent periods of human history where larger populations have been progressively supported by the cultivation of less land per person (Ellis et al. 2018), and it is against this that these earliest trends towards extensification must be reconciled. Part of this reconciliation can be found in the emergence of longer supply chains of trade that contributed to feeding early cities, but to this we may add the development of a new mosaic of land use, encompassing a wider variety of crops, that operated on different temporal cycles.

The period between the appearance of domesticated cereals that supported large Neolithic mega-sites like Çatalhöyük in the seventh millennium bc (Bogaard et al. 2017) and the rise of urbanism in the West Asia was around 4,000 years. In China, we might estimate this period to have been shorter, with some 1,500 years separating the end of domestication and the rise of urbanism (from ca 4000 to 2500 bc). One of the agricultural developments that emerged in this period, as emphasized by Sherratt (1999), was the introduction of commodity crops. Value was added in the transformation of these crops into dried fruits, oil, wine etc., increasing their longevity while reducing their bulk and facilitating their transportation as prestige goods. The first cities then emerged as part of this process, as centres with specialized social and economic functions, drawing in raw agricultural produce from their surrounding hinterland, transforming it into added value commodities, and redistributing the products to an increasingly large non-farming population (Trigger 2003; Sherratt 2011). As argued by Renfrew (1972) in his assessment of the development of Aegean social complexity, the new exploitation of crops including olive and grape created “a new flexibility in subsistence strategies, and […] the possibility of production specialization in single commodities” (Renfrew 1972, p 280; also; Margaritis 2013). In other words, agricultural commodity specialization occurred at the forefront of trends to craft specialization.

The emergence of perennial domesticates during these periods then provided a new basis for manufactured food commodities for exchange to be routed through cities, accompanying the general increase in trade of specialised craft items. This secondary agricultural revolution can be seen as a parallel development comparable with the specialization of animal secondary products such as wool, milk, and animal traction (Sherratt 1999).

In West Asia these developments first arose on the peripheries of urban Mesopotamia, but the exchange of such items soon came under the control of urban elites (Wengrow 2008), increasing their value by association with emergent high-ranking social classes, and their institutions and temples. The production of textiles was similarly a process that drew on raw produce from the periphery that was transformed in value by urban labour. As explored by McCorrsiton (1997) for Mesopotamia, this involved high levels of wool imported into cities alongside the more labour-intensive processing of flax. Flax for linen, wool sheep, and perennial crops all contributed to a new form of land use encompassing longer-term (perennial) production, what we might term investment agriculture, to differentiate it from the annual cropping, or sustainability agriculture, that focused on annual production of staple foods. This investment agriculture often relied on longer-term input of labour into land, relying on accepted norms of long-term land tenure, something that urban societies and states tended to enforce. In contrast to annual cereal and pulse crops, fruit and vines required longer-term investment with delayed returns. This created a tension between increasing the production of staple calories to support denser populations and allocating land for commodity crops, a tension well-documented from historical periods, e.g. the Islamic expansion of cotton production in Iran at the expense of further grain production (Bulliet 2009). However, the expansion of crop diversity through long-lived perennials, such as tree-fruits and textile crops, has been less thoroughly documented for the later Neolithic, Chalcolithic and Early Bronze Age.

The cultivation of perennial crops can be seen as establishing a new philosophy of land use, requiring longer-term investment, which included the transformation of crop products into value-added commodities for further exchange. Thus the cultivation of perennials can be argued to have been a prerequisite for some of the social changes that underpinned urbanization. These include developments in land ownership and rights of tenure associated with emergent elite bodies, resulting from increased social stratification. Indeed, some of the earliest written law codes in ancient Egypt, Mesopotamia, Israel (Ellickson and Thorland 1995) and China (Zhang 2014) enshrined ownership of agriculturally productive land.

In the case of China, archaeobotanical research has clearly documented the establishment of cereal agriculture in the Neolithic by ca 4500–4000 bc in both the Yangtze and Yellow River basins, but less attention has been paid to the diversification in arboreal domesticates. The evidence presented in this paper suggests that tree fruit cultivation had begun by ca 3000 bc and domestication was underway during the third millennium bc when urbanism emerged by the end of that millennium. The extent to which tree-fruit commodities, or other cash crops such as those for textiles (hemp, ramie, silk raised on mulberry orchards), were also tied to urban elite controls and redistribution requires further research, but in general we hypothesize similar processes of agricultural diversification towards perennial investment systems in China as in Mesopotamia.

## Electronic supplementary material

Below is the link to the electronic supplementary material.

Supplementary material 1 (CSV 67 KB)

Supplementary material 2 (CSV 51 KB)

Supplementary material 3 (CSV 17 KB)

Supplementary material 4 (CSV 31 KB)

Supplementary material 5 (CSV 51 KB)

Supplementary material 6 (CSV 23 KB)

Supplementary material 7 (CSV 19 KB)

Supplementary material 8 (CSV 10 KB)

Supplementary material 9 (CSV 6 KB)

Supplementary material 10 (CSV 30 KB)

Supplementary material 11 (CSV 472 KB)
